# Exosomes and Their Role in the Life Cycle and Pathogenesis of RNA Viruses

**DOI:** 10.3390/v7062770

**Published:** 2015-06-19

**Authors:** Harendra Singh Chahar, Xiaoyong Bao, Antonella Casola

**Affiliations:** 1 Departments of Pediatrics, University of Texas Medical Branch at Galveston, Galveston, TX 77555, USA; E-Mails: hachahar@utmb.edu (H.S.C.); xibao@utmb.edu (X.B.); 2 Sealy Center for Vaccine Development, University of Texas Medical Branch at Galveston, Galveston, TX 77555, USA

**Keywords:** exosomes, microvesicles, RNA virus, pathogenesis, miRNA, infection

## Abstract

Exosomes are membrane-enclosed vesicles actively released into the extracellular space, whose content reflect the physiological/pathological state of the cells they originate from. These vesicles participate in cell-to-cell communication and transfer of biologically active proteins, lipids, and RNAs. Their role in viral infections is just beginning to be appreciated. RNA viruses are an important class of pathogens and affect millions of people worldwide. Recent studies on Human Immunodeficiency Virus (HIV), Hepatitis C Virus (HCV), human T-cell lymphotropic virus (HTLV), and Dengue Virus (DENV) have demonstrated that exosomes released from infected cells harbor and deliver many regulatory factors including viral RNA and proteins, viral and cellular miRNA, and other host functional genetic elements to neighboring cells, helping to establish productive infections and modulating cellular responses. Exosomes can either spread or limit an infection depending on the type of pathogen and target cells, and can be exploited as candidates for development of antiviral or vaccine treatments. This review summarizes recent progress made in understanding the role of exosomes in RNA virus infections with an emphasis on their potential contribution to pathogenesis.

## 1. Introduction

Exosomes are lipid bilayer membrane-enclosed nano-sized (30–100 nm) vesicles with a density of 1.13–1.19 g/mL, secreted by virtually all cell types, and formed during the maturation of endosomes upon invagination and budding of the limiting membrane of late endosomes as intraluminal vesicles (ILVs) of multivesicular bodies (MVBs). Exosomes were first observed in the early 1980s in the culture media of reticulocytes [1,2,3]. In their study, Harding *et al.* (1983) reported that clathrin-coated, pit-mediated endocytosis led to enrichment of gold-labeled transferrin on vesicles inside non-lysosomal multivesicular endosomes [1]. They observed that these endosomes, commonly known as MVBs, fused with the plasma membrane and released their inner vesicles by exocytosis. Johnstone, in 1987, coined the term “exosomes” for such vesicles, which are released from cells by exocytosis [1]. Exosomes are released by almost all cell types and have also been confirmed in all bodily fluids such as blood, urine, saliva, breast milk, bronchial lavage, cerebral spinal fluid, and amniotic fluid [4,5,6,7,8,9,10,11,12,13,14,15,16,17,18,19]. In order to best adapt to the surrounding microcosm and execute their functions and duties, continuous communication, achieved through methods like direct cell–cell contact or transfer of secreted molecules, is essential for cells and tissues. Although initially it was proposed that secretion of exosomes was a mechanism of discarding cellular waste [16,20,21], in recent years exosomes have emerged as an important tool for intercellular communication through the transfer of biologically active proteins, lipids, and RNAs [22]. Exosomes have been implicated in normal as well as pathophysiological conditions, such as lactation, immune response, neuronal function, development and progression of liver disease, neurodegenerative diseases, cancer, and viral infections [16,22,23,24,25,26,27]. Exosome-mediated extracellular delivery of nucleic acids and proteins among virally infected and uninfected bystander cells have been shown to play an important role in viral pathogenesis and control of host immune responses to infection [28,29,30,31]. This certainly suggests a crucial role for exosomes in the viral life cycle and this review focuses on the important role exosomes play in the life cycle of RNA viruses with an emphasis on their potential contributions to pathogenesis.

## 2. Molecular Composition of Exosomes

Exosomes are essentially cytoplasm enclosed in a lipid bilayer with exposed external domains of transmembrane proteins. Exosomes may contain all types of biomolecules like proteins, carbohydrates, lipids, and also a nucleic acid signature of source of origin. New purification methods providing highly pure preparations of exosomes have allowed the use of proteomic and molecular techniques to understand the molecular composition of exosomes. The presence of cellular proteins in exosome preparations from various cellular sources has been analyzed by various methods like western blot, fluorescence-activated cell sorting (FACS), ELISA, and mass spectrometry [11,32,33,34,35,36,37,38,39]. Extensive analyses involving quantitative RT-PCR and RNA deep sequencing to identify RNA species that are present in exosomes have also been carried out [40,41,42,43,44,45,46]. These extensive and in-depth analyses revealed that a defined subset of cellular proteins, probably involved in exosome biogenesis and maybe in some common exosome functions, is targeted specifically to exosomes. This may include cytoskeletal components such as actin and actin-binding proteins, tubulin, and proteins involved in intracellular membrane fusions and transport such as Annexins and Rab (Ras (rat sarcoma) genes from the rat brain) proteins [47,48]. Molecules involved in signal transduction such as protein kinases (14-3-3) and heterotrimeric G proteins, heat shock proteins (HSPs), such as HSP70 and HSP90, and MHC class I and II molecules are also part of this defined but common set of exosome proteins. Tetraspanins including CD9, CD63, CD81, and CD82, as well as cytoskeletal components such as actin, are among the most abundant proteins in exosomes from virtually any cell type. Since exosomes are generated through invagination of late endosomes, a variety of host proteins that participate in vesicle formation and trafficking such as apoptosis linked gene (ALG), 2 interacting protein X (ALIX), and tumor susceptibility gene 101 (TSG101) are also incorporated into the exosomes [49]. All of these proteins have been considered as consensus markers for exosomes [50,51,52].

**Figure 1:**
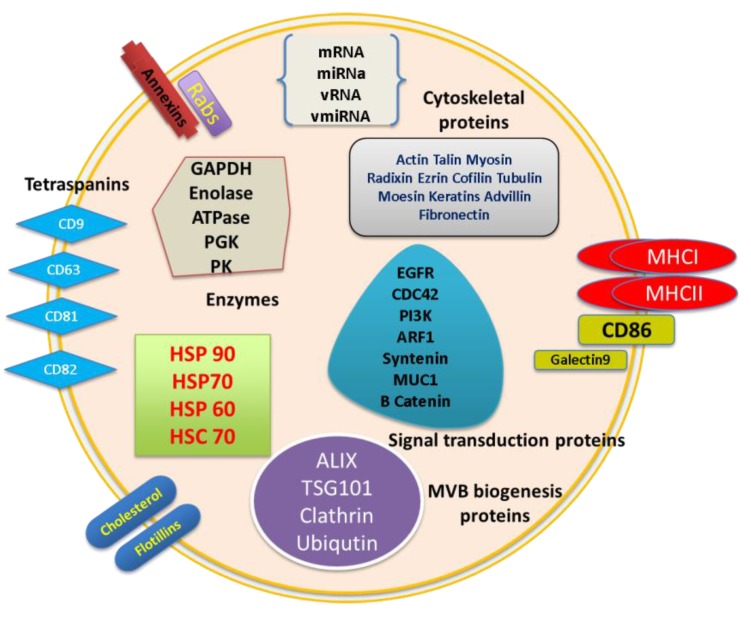
Structure and composition of exosomes. Exosomes contain a plasma membrane-derived phospholipid bilayer membrane. The composition depends on the cell type of origin, state of health of the host, and extracellular stimuli. Exosome contents include mRNA, miRNA, DNA, and proteins like annexins, tetraspanins, Alix, TSG101, MHC molecules, Rab proteins, cytoskeletal proteins, enzymes, and signal transduction proteins. GAPDH: Glyceraldehyde 3-phosphate dehydrogenase PGK: 3-phosphoglycerate kinase, PK: pyruvate kinase, EGFR: epidermal growth factor receptor, CDC42: cell division control protein 42, PI3k: phosphatidylinositide 3-kinases, ARF1: ADP-ribosylation factor 1, MUC1: Mucin 1, vRNA: viral RNA, vmiRNA: viral miRNA.

Exosomal lipid composition has also been characterized and exosomes are rich in sphingomyelin, gangliosides, phosphatidylserine, and cholesterol [53]. Nucleic acid signature is the other important component of exosomes and recent studies have focused on exosomal nucleic acid content. It has been demonstrated that exosomes carry biologically active mRNA, miRNA, other non-coding RNA, and a limited amount of DNA coding for ribosomal RNA [54,55,56,57,58]. However, the RNA and protein composition of exosomes varies in both quantity and type of molecules, depending on the origin and physiological/pathological state of the cells, suggesting that recruitment of RNA and protein into exosomes is a regulated process [55,59]. The exosome structure is graphically represented in Figure 1.

### 2.1. Biogenesis of Exosomes

Exosome biogenesis starts with the endocytosis and formation of early endosomes. The early endosome develops into the late endosome upon maturation, which is characterized by the formation of intraluminal vesicles (ILV) inside the lumen of the endosome. The ILVs, 30–100 nm in diameter, are formed by inward budding of the endosomal membrane, randomly engulfing portions of the cytosol and incorporating transmembrane and peripheral proteins into the invaginating membrane; this leads to formation of multivesicular bodies (MVBs) [5,60,61]. Although endocytosis and trafficking of plasma membrane receptors into MVBs is responsible for their degradation upon fusion with lysosomes [62], the fate of the MVBs may vary and not all MVBs are degraded in lysosomes, with a subset fusing with plasma membrane and resulting in generation of exosomes. The process of exosome biogenesis and cargo sorting is still not well understood and many studies suggest that the mechanisms of exosome biogenesis can be cell specific [63]. Exosomes are mainly secreted by two different mechanisms, constitutive release via the Trans-Golgi network and inducible release [64,65].

**Figure 2:**
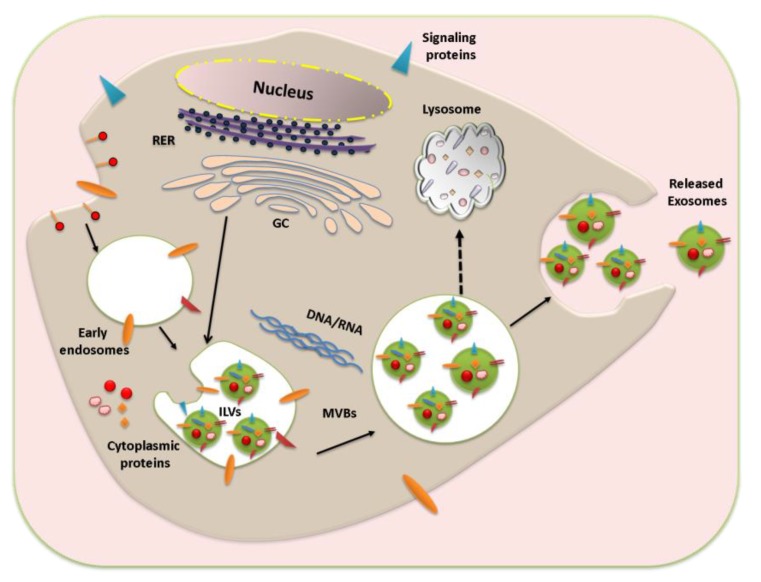
Schematic representation of exosome biogenesis and release. Exosome generation starts with early endosome formation during endocytosis. The membrane proteins are internalized through clathrin-coated vesicles and delivered to early endosomes. This leads to intraluminal vesicles (ILVs) formation by inward budding of the limiting membrane and multi vesicular bodies (MVBs) are formed. Upon maturation the exosome-filled MVBs are either sent to lysosomes for degradation or fused with the plasma membrane to release exosomes to the extracellular milieu. RER: Rough Endoplasmic Reticulum, GC: Golgi complex.

In the vesicle generation process, the endosomal sorting complexes required for transport 0 (ESCRT0) ubiquitinate proteins for MVB delivery and also recruit ESCRTI to endosomal membrane, which in turn recruits ESCRTII and ESCRTIII [66,67]. ESCRTIII mediates formation of polymeric filaments, which leads to membrane invaginations and eventually results in ILV formation [68]. The presence of ESCRT components in exosomes was identified using high throughput protein analysis methods, and downregulation of key components of ESCRT system abrogates ILV formation and release of exosomes [69]. Various studies also suggest ESCRT-independent mechanisms of exosome biogenesis and release. For example, in oligodendroglial cells exosome generation is regulated by the production of a lipid ceramide [70]. Recently, a CD63 tetraspanin-mediated mechanism of cargo sorting and ILV formation was reported, which is independent of ESCRT and ceramide [71]. Once the MVBs are formed, the soluble N-ethylmaleimide sensitive factor attachment receptor (SNARE) proteins and GTPases mediate their fusion with plasma membrane. Rab 35 has been recently shown to be part of the MVB docking to the membrane and depletion of Rab35 significantly decreased exosome release [72]. Although exosomes and ILVs are similar and generated through common mechanisms, cells have different populations of vesicles [47,73,74] and the mechanisms that contribute to exosome formation and cargo sorting within these vesicles is still not well understood. The process of exosome biogenesis is summarized in Figure 2.

### 2.2. Exosome Characterization

**Figure 3:**
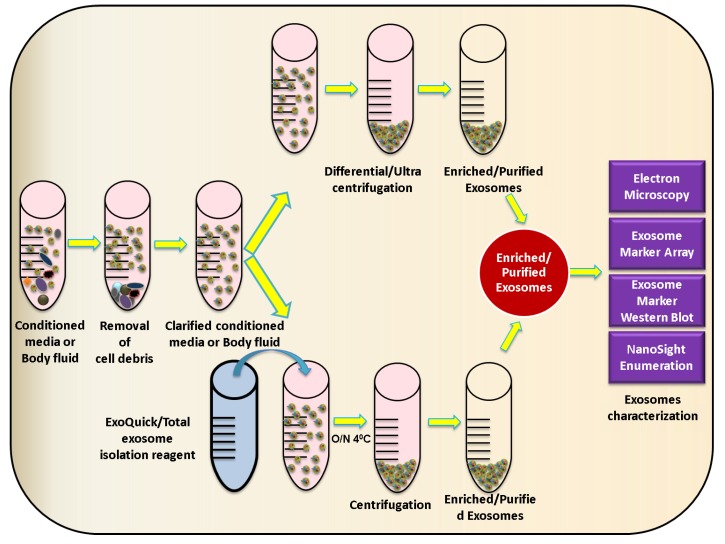
Schematic representation of exosome isolation strategies. Graphical representation of exosome isolation by both ultracentrifugation- and precipitation reagent-based isolation procedures, and analytical tools for exosome identification.

Various viruses like paramyxoviruses, HBV, HCV, rhabdoviruses, herpersviruses, filoviruses, and arenaviruses utilize or need the ESCRT pathway for their release [75,76,77]. Characterization and investigation of exosomes derived from virus-infected cells is a tough task as these vesicles often are of similar density and fall in the same size range as many of these viruses, making it necessary and at the same time challenging to separate the two populations. Conventionally, the exosomes are isolated from culture media or bodily fluids using a sequential sucrose-gradient ultracentrifugation procedure [78]. Other methods of exosome isolation/purification include microfiltration technologies, microfluidic devices [79,80,81,82], exosome precipitation reagents like ExoQuick™ (System Biosciences, Mountain View, CA, USA), and Total Exosome Isolation reagent (Life Technologies Grand Island, NY, USA), as well as antibody-coated magnetic bead-based immunopurification [38,83]. Similar sequential centrifugation and ultracentrifugation methods are used to purify many enveloped or non-enveloped viruses [84,85,86,87]. For instance, exosomes derived from HIV or HCV-infected cells cannot be readily distinguished or separated from infectious viral particles by conventional biophysical techniques as they share similar buoyant densities and sedimentation velocities [22,88,89,90]. Hence, when isolating exosomes from virus-infected cells, it becomes critically important to make sure that the pelleted material is exosomes and not virus particles. To address this, various exosome characterization methods have been developed including measurement and analysis of size distribution using NanoSight nanoparticle tracking analysis system, visualization of exosomes using electron microscopy, and immunoblot analysis of universal exosome protein markers like CD63, CD81, TSG101, Annexin5, ICAM1, FLOT1, and Alix (Figure 3).

**Figure 4:**
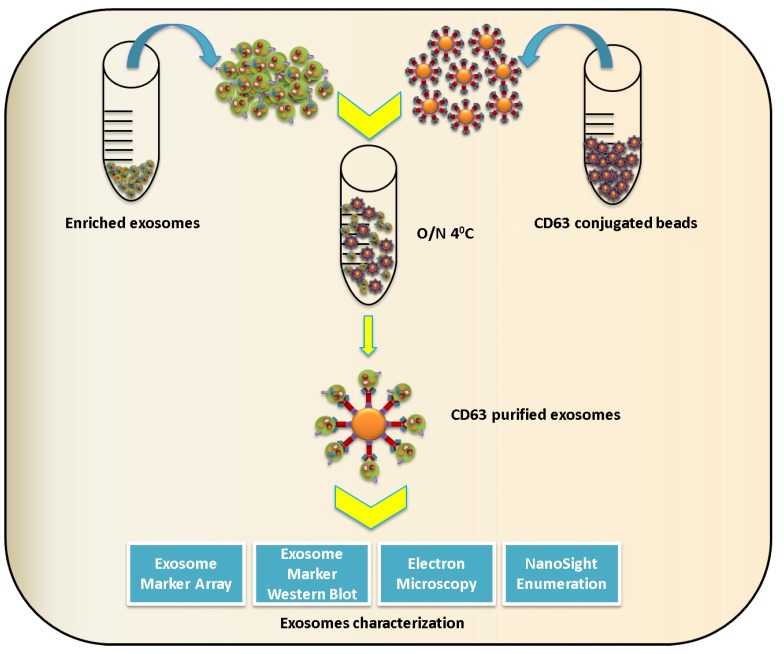
Schematic representation of exosome immune-isolation. To obtain exosomes free of contaminants, exosomes can be subjected to immunomagnetic selection using anti-CD63 antibody conjugated beads and then characterized by immunoblot, electron microscopy, and size determination.

When using immunoblot analysis, the exosome marker chosen for characterization should be selected with caution, as some viruses have been shown to incorporate exosome proteins. For example, proteomic studies utilizing liquid chromatography and tandem mass spectrometry (LC-MS/MS) found that influenza virus incorporates exosome markers ICAM1, Annexin A3, CD81, and CD9, while CD63 and ALIX were not present [91]. Similarly, exosomes and retroviruses share a variety of molecules like MHC-II [92,93], integrins (CD11a, CD18), co-stimulatory molecules (CD28, CD54), and complement neutralizing molecules (CD55, CD59) [5,35,94,95]. Various other host molecules/proteins acquired by enveloped viruses are reviewed in Cantin *et al.* [95]. Hence, the enriched exosomes isolated by ultracentrifugation or precipitation reagent should be further subjected to immunopurification methods like CD63 immunomagnetic bead isolation or other efficient virus purification strategies to obtain contamination-free populations of exosomes (Figure 4).

## 3. Transfer of Viral and Cellular Components by Exosomes and Their Role in Virus Replication and Transmission

Although the field of exosomes and their contribution to replication and pathogenesis of RNA viruses remain largely unexplored, a few RNA viruses have been investigated, such as HIV-1, HTLV-1, HCV, and Dengue virus. Findings from these studies have demonstrated that exosomes released from virus-infected cells contain a variety of viral and host cellular factors that are able to modulate recipient host cell responses and lead to the establishment of productive infection.

### 3.1. Exosomes as Carriers of Virus and Host RNA Species

It has been reported that exosomes carry various cellular regulatory RNAs, including miRNAs, sncRNAs, and siRNAs [96]. Exosomes derived from virus-infected cells have been shown to carry viral components including viral mRNA, miRNA, and genomic RNA, as well as genetic regulatory elements. Among the RNA viruses, HIV-1 was the first one to be studied somewhat in detail in the context of modulation of exosome formation. Exosomes derived from HIV-1 infected cells or patients with HIV infection incorporate the viral transactivating response (TAR) element transcribed from the integrated provirus, which has been suggested to enhance HIV replication in the recipient cells via downregulation of apoptotis [42]. Unspliced HIV-1 RNA species are recruited to exosomes and the presence of a stretch of sequences within the 5' end of the Gag p17 open reading frame is sufficient for this recruitment, while single- or double-spliced HIV-1 RNA is not incorporated into exosomes. The incorporation of genomic HIV-1 RNA in exosomes is further increased if the producer cells express HIV-1 defective for viral genome packaging [97].

Exosome-like microvesicles isolated from serum during infection with human Pegivirus (an RNA virus within the *Pegivirus* genus of the *Flaviviridae* previously known as Hepatitis G virus) have been shown to carry viral RNA and to be able to transmit viral RNA to peripheral blood mononuclear cells *in vitro*, resulting in productive infection [98]. Exosomes released from HCV-infected cells contain HCV RNA, which can be successfully transferred to dendritic cells to establish productive infection [99]. Two subsequent studies confirmed that exosomes derived from HCV-infected hepatocytes contain the complete HCV genome and they have the ability to mediate transfer of replication-competent subgenomic HCV RNA to permissive naive cells, leading to viral RNA replication and productive infection [100,101]. Exosomes derived from HCV-infected patients have also been found to contain negative sense HCV RNA (replication competent viral RNA), in association with Ago2, HSP90, and miR-122 [102].

A recent study reported that exosomes derived from Human T-lymphotropic virus type 1 (HTLV-1)-infected cells contain the Tax, HBZ, and Env gene mRNA transcripts, suggesting that exosomes can serve as vehicles to deliver functional HTLV-1 mRNA to recipient cells [103].

Exosomes secreted from HIV-1-infected primary alveolar macrophages carry viral microRNAs vmiR88, vmiR99, and vmiR-TAR; these miRNAs have also been detected in exosomes purified from the sera of HIV-infected individuals. Viral microRNAs vmiR88 and vmiR99 were shown to stimulate signaling in macrophages, resulting in robust release of TNFα through macrophage endosomal TLR8 [104]. Expression of HIV Nef protein in macrophage-like cells results in selective recruitment of 47 miRNAs into exosomes, with two miRNAs selectively retained in the same cells [105], suggesting that modulation of exosomal RNA composition during a viral infection is a regulated process and that specific mechanisms exist to recruit or retain specific miRNAs/RNAs. Astrocytes exposed to a combination of HIV protein Tat and opiate drugs secreted exosomes with elevated levels of miR-29b. In human neurons exposed to miR-29b-enriched exosomes, platelet-derived growth factor-B expression was repressed and neuron viability was correspondingly decreased [106]. The presence of Ago2, an essential protein of RNA-induced silencing complex (RISC) for mediating miRNA-targeted gene suppression, and cellular miRNAs in exosomes secreted from HTLV-1-infected cells suggested that HTLV-1 could manipulate mRNA translation in recipient cells [107].

### 3.2. Exosomes as Viral Protein Carriers

Exosomal protein composition varies depending on cell type and disease state. Several RNA viruses have been shown to modulate not only host cell protein composition of exosomes but also to recruit their own proteins into exosomes. Exosomes from HIV-1-infected cells have been shown to incorporate both Gag [108] and Nef proteins [109,110]. The latter is incorporated into exosomes upon anchoring into lipid raft microdomains through its N-terminal myristoylation and a stretch of basic amino acids residing in its alpha-helix-1 [111], as well as upon interaction with the host cell protein Mortalin [112].

Similar to HIV, the HTLV-1 transactivator protein Tax, a critical factor for proliferation and transformation of T cells, is incorporated in exosomes secreted from virally-infected cells [103]. Several cellular proteins also seem to be recruited to exosomes in a Tax-dependent manner, many of them involved in protein synthesis and antigen presentation [103]. Exosomes derived from HCV-infected cells have been shown to carry HCV core protein, in addition to apolipoproteins ApoE and ApoB [101]. In HCV-positive patients, the cellular membrane protein CD81 has been shown to associate with the envelope glycoprotein E2. Extracellular release of E2-CD81 within microvesicles is associated with increased virus fusing ability and infectivity of naive cells [113]. Table 1 enlists the protein and RNA species of RNA viruses recruited to exosomes.

**Table 1:** Viral protein and RNA species present in exosomes derived from RNA virus-infected cells.

	Protein and RNA Species of RNA Viruses Present in Exosomes
**Viral Proteins**	**HIV:** Nef and Gag proteins **HCV:** HCV core protein **HTLV-1:** Transactivator protein Tax
**Viral RNA and microRNA**	**HIV:** HIV-1 transactivating response (TAR) element RNA, microRNAs vmiR88, vmiR99 and vmiR-TAR, unspliced HIV-1 RNA species, **HCV:** HCV genomic RNA **HTLV-1:** HTLV-1 Tax, HBZ, and Env gene mRNA transcripts

### 3.3. Role of Exosomes in Pathogenesis

Immature dendritic cells capture HIV-1 and can transfer these captured HIV-1 particles to T cells. Wiley and Gummuluru, back in 2006, reported that exosomes derived from HIV-1 containing immature dendritic cells can transfer HIV-1 to T cells without *de novo* infection. Exosomes isolated with HLA-DR-1-conjugated magnetic beads from the supernatant of DCs exposed to HIV-luc reporter viruses incubated with Jurkat T cells resulted in productive infection of cells. They also reported that endocytosed virus particles were the main contributors to exocytosed virus fraction, as treatment of virus-exposed DCs with trypsin had no or a negligible effect on the amount of virus particles precipitated by HLA-DR-1-conjugated magnetic beads [114].

Although the role of exosomes in HIV infection has not yet been fully understood, increasing evidence suggests that exosomes facilitate both enhancement and inhibition of infection and replication, depending upon the cells of origin. For instance, exosomes derived from HIV-infected cells have been shown to contain the HIV coreceptors CCR5 and CXCR4, and transfer of these coreceptors to uninfected, non-permissive cells may enhance susceptibility to HIV infection [115,116]. In a recent study, Kadiu *et al.* reported that a portion of HIV virions shed from monocyte-derived macrophages is associated with exosomal aggregates and these entrapped virions demonstrate improved infectivity toward CD4^+^ target cells, compared to purified HIV-1 virus particles [117]. HIV-infected and viremic individuals exhibit elevated levels of plasma cytokines. Many cytokines have been found to be markedly enriched in exosomes from HIV-positive individuals relative to negative controls and exposure of naive peripheral blood mononuclear cells to exosomes purified from HIV-positive patients induces CD38 expression on naive and central memory CD4^+^ and CD8^+^ T cells, probably contributing to inflammation and viral propagation via bystander cell activation [118].

HIV accessory protein negative factor (Nef) is one of the earliest and most abundantly expressed viral proteins. Nef is also released in exosomes. Lenassi *et al.* (2010) reported that Nef not only stimulates its own export through exosomes, but these Nef exosomes also facilitate the depletion of CD4^+^ T cells that is a hallmark of acquired immunodeficiency syndrome (AIDS) [109]. In fact HIV Nef, through exosomes, induces the activation of resting CD4^+^ T lymphocytes, rendering these quiescent CD4^+^ T lymphocytes permissive to HIV-1 replication and thus stimulating viral spread [119]. HIV-1 Nef promotes viral replication and pathogenesis by mediating depletion of CD4 and MHC-I molecules. Nef binds to the cytosolic tail (CT) of CD4 and MHC-I and disrupts the intracellular trafficking of these proteins targeting them to multivesicular bodies (MVBs), and ultimately to lysosomes for degradation. In a recent study Carvalho *et al.* (2014) reported that exosomes secreted by CD4^+^ T cells, but not CD4^−^ T cells, efficiently inhibit HIV-1 infection *in vitro*, suggesting that exosomal CD4 can bind to envelope proteins of HIV-1, hindering virus interaction with target cells and eventually reducing the infection [110]. They also showed that this effect could be reversed by depleting CD4 exosomes released by CD4^+^ T cells expressing Nef. The remaining exosomes have a reduced capacity to inhibit HV-1 infection *in vitro* [110]. HIV Nef also modulates exosomal miRNA composition, further suggesting a role for exosomes in HIV pathogenesis and viral replication [110].

Exosomes play an important role in HTLV-1 infection as well, probably by delivering functional HTLV-1 Tax protein, proinflammatory mediators, and viral mRNA transcripts of Tax, HBZ, and Env proteins. Along with other host proteins, major histocompatibility complex (MHC) class I A and class I E precursor were also identified in exosomes derived from HTLV-1 infected cells. The addition of C81 cell-derived exosomes (containing Tax protein) to myeloid dendritic cells resulted in a significant increase in the levels of IL-2, IL-5, and IL-6 cytokines. In fact, cell-free Tax could induce IL-10, IL-12, IL-17A, IFN-γ, and G-CSF secretion from dendritic cells. The findings of Jaworski *et al.* (2014) suggest that exosomes play a crucial role in signal transduction and may contribute to pathogenesis of HTLV-1 infection [103].

Human cytidine deaminase APOBEC3G (A3G) is part of a cellular defense system against HIV-1 as well as other retroviruses. In a recent study, Khatua *et al.* (2009) found that APOBEC3G secreted by cells in exosomes can confer resistance to both defective and wild-type HIV-1 infection in exosome recipient cells [120]. Esser *et al.* demonstrated that CD45, CD86, and MHC Class II molecules present in exosomes derived from HIV-infected cells may help in silencing immune response, therefore favoring virus replication [121]. Exosomes derived from HIV-1 infected CD8^+^ T cells suppressed replication of both CCR5- and CXCR4-tropic HIV-1 strains *in vitro* by inhibiting HIV-1 transcription in both acute and chronic models of infection [122]. Exosomes can not only transmit HCV to naive cells but also offer some degree of protection from HCV neutralizing antibodies. By making use of transmission electron microscopic imaging, Liu *et al.* demonstrated that HCV was present in both exosome-free and exosome-associated forms and the association with exosomes conferred the ability to resist anti-HCV antibody mediated neutralization, suggesting that HCV may utilize transmission via exosomes as an immune evasion mechanism [101,123].

Recently a new set of proteins called IFN inducible transmembrane proteins 1, 2, and 3 (IFITM1, 2, and 3) have been shown to display antiviral activities induced by IFN, conferring host cells resistance to various viral pathogens [124]. In their study Zhu *et al.* (2015) found that the IFITM3 protein level in host cells inversely correlates with their susceptibility to Dengue Virus-2 (DENV-2) infection [124]. Exosomes derived from HUVEC or HepG2 cells contain IFITM3 protein and can transfer this protein to neighboring cells. Investigating the functional aspect of this exosome-mediated transfer of IFITM3, they found that in recipient cells DENV-2 infection was effectively suppressed by the IFITM3-containing exosomes in a dose-dependent manner. The authors suggested that the IFITM3-containing exosomes did not affect the binding or post-entry steps during DENV-2 infection, but reduced the penetration of DENV-2 into cells, demonstrating an important role for exosomes in DENV-2 infection [124]. There is also evidence that some viruses harness exosomes to avoid immune recognition in the bloodstream or as reservoirs of virus latency. Hepatitis A virus (HAV), a non-enveloped virus, was found to be encapsulated into vesicles derived from endosomal membranes [47]. These enveloped HAV particles were fully infectious. They resembled exosomes and their biogenesis was dependent on ESCRTs and ESCRT effectors [47]. Their membrane cloak protected them from antibody neutralization and virus-specific antibodies appear only after 3–4 weeks of infection.

## 4. Potential Applications in Viral Infections

Exosomes appear to be an important tool of intercellular communication, as discussed above. However, their further use in various other processes is also being evaluated. The exosomes can be used as a diagnostic marker, as vaccines, and as a drug delivery vehicle for targeted or systemic delivery. Since exosomes have been detected in all bodily fluids, can be easily purified, and have a composition that varies in normal and diseased conditions, they can be exploited as diagnostic markers of diseases. However, the use of exosomes as a diagnostic marker for viral infection has not yet been explored adequately.

Targeted delivery is another area where the potential of exosomes to carry therapeutic cargo to specific organs or tissues is being evaluated. Expression of receptor-specific ligand molecules on the exosome surface through genetic engineering can transform exosomes into potent delivery vehicles that can deliver a drug/siRNA/miRNA based therapeutic moiety to cells or tissues of choice. In fact, the ability of exosomes to deliver therapeutic moiety or genetic material can be further improved by incorporating selected viral proteins into exosomes as virus-encoded envelope proteins exhibiting superior binding and entry specificity (reviewed in [125]). For instance, exosomes engineered to express a 29-mer peptide derived from the rabies virus glycoprotein (RVG), which specifically binds to acetylcholine receptors expressed on the brain cells, were exploited by Alvarez-Erviti *et al.* (2011) to transport small interfering RNAs to the brain [126]. The immature dendritic cells (DCs) were transfected with plasmids encoding exosomal protein Lamp2b, fused with the 29-mer RVG peptide. Exosomes were purified from DC cultures, loaded with GAPDH or BACE-1 siRNA, and injected intravenously through tail vein injection. The targeted delivery resulted in specific knockdown of GAPDH and BACE-1 in the mouse brain [127]. In other studies, DCs were transduced with adenoviral vector to express Interleukin (IL)-10, IL-4, or FasL, and the engineered exosomes were used to treat autoimmune disorders and inflammatory diseases (reviewed in [128]). Similar methods can be utilized to engineer exosomes to deliver siRNAs to control viral infections such as West Nile Virus or other viral infections of the brain or other organs like the liver or lungs. Exosomes are also being evaluated as vaccines in the field of infectious diseases. Aline *et al.* (2004), investigated the efficacy of DC2.4 cell line-derived exosomes to mount a protective immune response against toxoplasmosis [129]. They found that *Toxoplasma gondii*-pulsed DC-derived exosomes transferred to the spleen, elicited a strong systemic Th1-modulated *Toxoplasma*-specific immune response *in vivo*, and were able to protect the animals against *Toxoplasma* infection [129]. This suggests that exosomes can also be used for immunoprophylaxis against viral pathogens; however, systemic studies need to be conducted to evaluate the therapeutic or protective role engineered exosomes can play in the field of infectious diseases. Exosomes offer many advantages, including but not limited to being natural transport body vehicles of antigens and signals between cells, providing a stable environment for nucleic acids and proteins by protecting them from DNase, RNase, and proteinases, efficient association/interaction with antigen-presenting cells, and offering better molecular distribution capabilities as are present in all bodily fluids [125,130].

## 5. Conclusions

Various studies have demonstrated that exosomes are crucial intercellular communication channels and highlighted their potential role in viral transmission and modulation of immune responses, as viruses exploit the exosomal pathway for their assembly/budding, transfer of viral RNAs, and suppression of immune activation. In addition, exosomes could be utilized as diagnostic markers in viral infections and for targeted drug delivery. Since exosome research related to viral infections is still in an early stage, more studies are required to decipher the interplay between exosome biology and viruses, as a comprehensive understanding of exosome biology and its involvement in viral infections would permit the development of new strategies to interfere with viral replication and disease development.
